# Customized 3D-Printed Wrist-Thumb Orthoses for De Quervain’s Tenosynovitis: A Prospective Observational Study

**DOI:** 10.7759/cureus.111250

**Published:** 2026-06-21

**Authors:** Md. Israt Hasan, Syed Mozaffar Ahmed, Mohammed Emran, Taufiq Morshed, Fatema Newaz, Shamim Ahmed Deowan, Md. Atiquzzaman

**Affiliations:** 1 Department of Physical Medicine and Rehabilitation, Sher-E-Bangla Medical College, Barishal, BGD; 2 Department of Physical Medicine and Rehabilitation, Bangladesh Medical University, Dhaka, BGD; 3 Department of Physical Medicine and Rehabilitation, Khwaja Yunus Ali Medical College, Sirajganj, BGD; 4 Department of Orthopedic Surgery, National Institute of Traumatology and Orthopedic Rehabilitation, Dhaka, BGD; 5 Department of Physical Medicine and Rehabilitation, Kumudini Women's Medical College and Hospital, Tangail, BGD; 6 Department of Robotics and Mechatronics Engineering, University of Dhaka, Dhaka, BGD; 7 Department of Physical Medicine and Rehabilitation, Kurmitola General Hospital, Dhaka, BGD

**Keywords:** 3d printing, de quervain’s tenosynovitis, patient satisfaction, rehabilitation medicine, wrist-thumb orthosis

## Abstract

Background: Wrist-thumb orthoses are commonly used in the conservative management of De Quervain’s tenosynovitis; however, conventional fabrication techniques often limit anatomical fit and patient comfort. Three-dimensional (3D) printing enables patient-specific orthosis design and may improve usability, particularly in resource-limited rehabilitation settings.

Objective: To evaluate the feasibility, clinical outcomes, and patient satisfaction associated with customized 3D-printed wrist-thumb orthoses in individuals with De Quervain’s tenosynovitis.

Methods: This prospective observational study included 40 patients with clinically diagnosed De Quervain’s tenosynovitis, of whom 37 completed a six-week follow-up. Participants received either a conventional thumb spica splint or a customized 3D-printed wrist-thumb orthosis based on feasibility and availability. Pain intensity and upper limb functional status were evaluated using the Visual Analogue Scale (VAS) and the Quick Disabilities of the Arm, Shoulder and Hand (QuickDASH) instrument at baseline, two weeks, four weeks, and six weeks. Satisfaction with the 3D-printed orthosis was measured using the Quebec User Evaluation of Satisfaction with Assistive Technology (QUEST 2.0; MARCH Laboratory (Modeling for Assistive Technology and Community Living), Centre for Interdisciplinary Research in Rehabilitation of Greater Montreal (CRIR), Montreal, Quebec, Canada). Only descriptive statistical analyses were performed.

Results: Both groups demonstrated substantial reductions in pain and functional disability over time. In the conventional group, mean VAS decreased from 81.25 at baseline to 14.32 at six weeks, while QuickDASH improved from 76.25 to 16.84. In the 3D-printed group, mean VAS decreased from 75.60 to 10.07, and QuickDASH improved from 72.15 to 11.34 over the same period. Numerically greater improvements were observed in the 3D-printed group. QUEST scores ranged from 3.0 to 4.5, indicating high levels of patient satisfaction. No device-related adverse events were reported.

Conclusion: Customized 3D-printed wrist-thumb orthoses are feasible and associated with improvements in pain, functional outcomes, and patient satisfaction in patients with De Quervain’s tenosynovitis. Findings should be interpreted as descriptive trends, and further studies with larger samples and inferential analyses are warranted.

## Introduction

An orthosis is an externally applied device designed to support, align, protect, or correct musculoskeletal structures; restrict or assist movement; and facilitate functional activities such as weight-bearing. Orthoses are integral to orthopedic and physical medicine and rehabilitation practice and are widely used in the management of fractures, sprains, post-arthroplasty rehabilitation, tendinopathies, and neurological disorders [1,2]. Their effectiveness depends not only on biomechanical function but also on comfort, durability, and patient adherence [1].

Conventional orthosis fabrication is predominantly manual, requiring skilled craftsmanship and considerable production time. Achieving precise anatomical customization is often challenging, particularly when repeated adjustments are necessary. Manually fabricated orthoses may also be prone to structural limitations, including weakened stitched seams or deterioration of fastening systems such as Velcro (VELCRO® Brand Fasteners, Velcro Companies, Manchester, New Hampshire, USA), which can compromise durability and long-term use [3,4]. Moreover, traditional manufacturing methods offer limited capacity to systematically integrate ergonomic principles, biomechanical design rules, and functional considerations into the final orthosis design [4].

Three-dimensional (3D) printing has emerged as an innovative alternative to traditional orthosis manufacturing techniques. Since its introduction in the mid-1980s, this form of additive manufacturing has enabled the layer-by-layer creation of physical objects from digital designs, differing fundamentally from conventional subtractive methods [5]. Although initially adopted for rapid prototyping, advances in materials, software, and declining costs have expanded its application into various medical fields, including orthopedics and rehabilitation medicine [5-7]. Using computer-aided design and patient-specific digital modeling, 3D printing enables the fabrication of customized orthoses with precise control over geometry, thickness, and fit [4-6]. This approach allows rapid modification based on clinical feedback and may improve comfort, usability, and adherence [4-6].

Ergonomics is a key determinant of patient satisfaction and prolonged orthosis use [8]. Poorly fitting or uncomfortable devices are associated with reduced compliance and suboptimal clinical outcomes. Studies have demonstrated that commercially available prefabricated orthoses often fail to adequately meet patient expectations regarding fit and comfort [5]. In contrast, 3D-printed orthoses can be tailored to individual anatomy, offering improved conformity, reduced bulk, and enhanced ventilation. These advantages suggest that 3D printing technology has the potential to significantly improve orthotic care within rehabilitation settings [5,6].

De Quervain’s tenosynovitis is a frequently encountered condition involving the first dorsal compartment of the wrist, characterized by restricted movement of the abductor pollicis longus and extensor pollicis brevis tendons [9]. Although traditionally considered an inflammatory condition, histopathological studies suggest that it is primarily a degenerative tendinopathy characterized by myxoid degeneration, tendon sheath thickening, and fibrocartilaginous metaplasia. Repetitive thumb movements, forceful grasping, and occupational or recreational overuse are recognized contributing factors that may lead to increased friction and stenosis within the first dorsal compartment [9]. The disorder was initially described by Fritz de Quervain in 1895 and has since been further detailed in subsequent literature [9,10]. It is commonly associated with thickening of the extensor retinaculum, which contributes to tendon entrapment and inflammatory changes. Epidemiological studies report a prevalence of approximately 0.5% in men and 1.3% in women, with higher incidence among individuals engaged in repetitive or forceful manual activities [11-13].

Clinically, individuals commonly report pain and tenderness over the radial styloid region, which may extend toward the thumb or forearm. Associated features can include swelling, focal tenderness, and occasionally crepitus, while Finkelstein’s test is often positive [13,14]. The diagnosis is largely clinical, relying on a detailed history and physical examination [12,13]. De Quervain’s tenosynovitis is also considered a work-related musculoskeletal condition, with recognized risk factors such as repetitive thumb use, forceful gripping, and prolonged wrist ulnar deviation [13].

Management includes both operative and non-operative approaches, with conservative treatment considered first-line [15]. Non-operative measures include rest, activity modification, non-steroidal anti-inflammatory drugs, therapeutic exercises, and splinting. Immobilization of the wrist and thumb using a static splint is commonly recommended for several weeks, particularly in early or mild cases, as it helps minimize mechanical irritation and supports symptom relief [15,16]. Splinting limits wrist and thumb motion, reduces mechanical irritation, and promotes tendon healing while allowing functional use of the remaining digits. Although various splint designs and materials have demonstrated comparable clinical outcomes, limitations related to fit, comfort, and durability remain [16].

Integrating 3D printing technology into wrist-thumb orthosis fabrication offers an opportunity to address these limitations by producing patient-specific devices that optimize ergonomics and satisfaction [17]. This personalized approach aligns with contemporary rehabilitation principles and provides the rationale for evaluating customized 3D-printed orthoses in the management of De Quervain’s tenosynovitis. In low-resource settings such as Bangladesh, access to customized orthotic fabrication remains limited, highlighting the need for scalable and cost-effective solutions.

## Materials and methods

Study design and participants

A prospective observational study was carried out in the Department of Physical Medicine and Rehabilitation at Bangladesh Medical University (BMU), Dhaka, over a duration of one year. Patients attending the outpatient department with a clinical diagnosis of De Quervain’s tenosynovitis were consecutively enrolled. Diagnosis was based on pain over the radial styloid process, tenderness over the first extensor compartment, and a positive Finkelstein’s test. Participants received either a conventional thumb spica splint or a customized 3D-printed wrist-thumb orthosis based on feasibility, including availability of 3D printing resources. No randomization or controlled allocation was performed.

Inclusion criteria

Patients aged 18 years and above with clinically diagnosed De Quervain’s tenosynovitis were included in the study.

Exclusion criteria

Individuals with inflammatory joint disorders, gout, prior wrist trauma, a history of local corticosteroid injection, radiculopathy, or carpal tunnel syndrome were excluded. Patients with local infection or skin lesions in the affected area, as well as those who declined participation, were also not included in the study.

Sample size and sampling technique

The sample size was estimated using the standard formula for a single population proportion [18]: $n = \frac{Z^2 \times p(1-p)}{d^2}$, where n represents the required sample size, Z is the standard normal deviate corresponding to a 95% confidence interval (1.96), p is the estimated proportion, and d is the allowable margin of error. In the absence of reliable local epidemiological data for De Quervain’s tenosynovitis, a conservative estimate of p = 0.5 was used to ensure maximum variability, while the precision level was set at d = 0.15. Using these assumptions, the calculated minimum sample size was approximately 43 participants. Considering feasibility constraints and the exploratory nature of the study, 40 participants were ultimately included. Participants were recruited purposively from the outpatient department. Due to feasibility constraints and differential availability of customized orthoses, unequal group sizes were observed between the conventional and 3D-printed orthosis groups. Follow-up data were available for 37 participants.

Assessment tools

Pain intensity was assessed using the Visual Analogue Scale (VAS) [19]. Functional status was evaluated using the Quick Disabilities of the Arm, Shoulder and Hand (QuickDASH) questionnaire [20]. Patient satisfaction in the 3D-printed orthosis group was assessed using the Quebec User Evaluation of Satisfaction with Assistive Technology version 2.0 (QUEST 2.0; MARCH Laboratory (Modeling for Assistive Technology and Community Living), Centre for Interdisciplinary Research in Rehabilitation of Greater Montreal (CRIR), Montreal, Quebec, Canada) [21].

The Quebec User Evaluation of Satisfaction with Assistive Technology (QUEST 2.0) questionnaire was used with permission from the original developers. Scores were reported as mean item scores ranging from 1 to 5. Formal permission was also obtained to translate the questionnaire into Bangla for use in this study. The translated version has not yet undergone formal psychometric validation.

Data analysis

The collected data were analyzed using descriptive statistical methods, with results presented as mean values and standard deviations. Individual-level datasets were not preserved in a retrievable format for reanalysis; therefore, inferential statistical comparisons were not performed.

Ethical considerations

Ethical clearance for this study was granted by the Institutional Review Board of Bangabandhu Sheikh Mujib Medical University (BSMMU), currently Bangladesh Medical University (BMU), Dhaka, Bangladesh (Approval No. BSMMU/2022/8034; dated: August 17, 2022). Written informed consent was obtained from all participants prior to enrolment and data collection.

## Results

A total of 40 participants were recruited for the study, of whom 37 completed the follow-up assessments. Among these, 20 patients were allocated to the conventional thumb spica splint group, while 17 received the customized 3D-printed wrist-thumb orthosis. Three participants in the 3D-printed group were lost to follow-up. Reasons for loss to follow-up included non-compliance and inability to attend scheduled visits. Baseline sociodemographic characteristics and clinical measures are summarized in Table 1.

**Table 1:** Sociodemographic data and baseline measurements Values are presented as n (%) or mean ± standard deviation. VAS, Visual Analogue Scale; QuickDASH, Quick Disabilities of the Arm, Shoulder and Hand questionnaire; SD, standard deviation.

	Conventional Splint (n=20)	3D-Printed Splint (n=17)
Sociodemographic data		
Age (years)	39.50 ± 12.32	44.50 ± 13.43
Female (%)	75 (15)	70.59 (12)
Occupation		
Housewife	9	11
Teacher	4	3
Manual laborer	3	1
Office worker	4	2
Hand involvement		
Right hand	16	13
Left hand	4	4
Baseline measurements		
QuickDASH	76.25 ± 8.06	72.15 ± 5.09
VAS	81.25 ± 10.08	75.60 ± 7.07

Baseline characteristics

The mean age of participants was 39.50 ± 12.32 years in the conventional splint group and 44.50 ± 13.43 years in the 3D-printed splint group. Females constituted the majority in both groups, accounting for 75% of the conventional group and 70.59% of the 3D-printed group. The most common occupation was housewife, followed by teacher, office worker, and manual laborer. Right-hand involvement was more frequent than left-hand involvement in both groups. At baseline, mean Visual Analog Scale (VAS) pain scores were 81.25 ± 10.08 in the conventional splint group and 75.60 ± 7.07 in the 3D-printed splint group. Mean QuickDASH scores at baseline were 76.25 ± 8.06 and 72.15 ± 5.09, respectively, indicating substantial pain and functional limitation at study entry.

Pain and functional outcomes

Changes in pain intensity and functional status over time are presented in Table 2. In the conventional thumb spica group, mean VAS scores decreased progressively from 81.25 ± 10.08 at baseline to 46.03 ± 3.34 at two weeks, 16.03 ± 2.24 at four weeks, and 14.32 ± 2.01 at six weeks. Correspondingly, mean QuickDASH scores improved from 76.25 ± 8.06 at baseline to 28.73 ± 16.33 at two weeks, 18.94 ± 11.94 at four weeks, and 16.84 ± 9.05 at six weeks.

**Table 2:** Changes in pain and functional outcomes over time Changes in pain and functional outcomes over time in the conventional and 3D-printed orthosis groups. Values are presented as mean ± standard deviation (SD). VAS, Visual Analogue Scale; QuickDASH, Quick Disabilities of the Arm, Shoulder and Hand questionnaire; SD, standard deviation.

Time Point	Conventional Group (n=20) VAS (Mean ± SD)	Conventional QuickDASH (Mean ± SD)	3D-Printed Group (n=17) VAS (Mean ± SD)	3D-Printed QuickDASH (Mean ± SD)
Baseline	81.25 ± 10.08	76.25 ± 8.06	75.60 ± 7.07	72.15 ± 5.09
2 weeks	46.03 ± 3.34	28.73 ± 16.33	51.09 ± 6.00	36.22 ± 7.35
4 weeks	16.03 ± 2.24	18.94 ± 11.94	11.07 ± 8.01	14.03 ± 9.11
6 weeks	14.32 ± 2.01	16.84 ± 9.05	10.07 ± 6.23	11.34 ± 7.75

In the 3D-printed thumb spica group, mean VAS scores decreased from 75.60 ± 7.07 at baseline to 51.09 ± 6.00 at two weeks, 11.07 ± 8.01 at four weeks, and 10.07 ± 6.23 at six weeks. Mean QuickDASH scores improved from 72.15 ± 5.09 at baseline to 36.22 ± 7.35 at two weeks, 14.03 ± 9.11 at four weeks, and 11.34 ± 7.75 at six weeks. Overall, both groups demonstrated substantial reductions in pain and functional disability over the follow-up period.

Patient satisfaction with 3D-printed orthosis

Patient satisfaction was evaluated exclusively in the 3D-printed orthosis group using the Quebec User Evaluation of Satisfaction with Assistive Technology (QUEST) version 2.0. Scores for the device, service, and overall satisfaction domains are summarized in Table 3.

**Table 3:** The calculated QUEST score User satisfaction scores for the 3D-printed orthosis based on the QUEST 2.0 assessment. Values are presented as scores on a five-point Likert scale. QUEST, Quebec User Evaluation of Satisfaction with Assistive Technology.

Cases	Device	Service	Total
Case 1	3.5	4.5	4
Case 2	4	3	3.5
Case 3	4.5	3.5	4
Case 4	4.5	4	4.25
Case 5	4	3.5	3.75
Case 6	3.5	3.5	3.5
Case 7	4.5	3.5	4
Case 8	3	4.5	3.75
Case 9	4	3	3.5
Case 10	3.5	4.5	4
Case 11	3	4.5	3.75
Case 12	4	4	4
Case 13	3.5	3	3.25
Case 14	4.5	4.5	4.5
Case 15	4	4.5	4.25
Case 16	3	3	3
Case 17	4.5	3.5	4

Total QUEST scores ranged from 3.0 to 4.5, indicating generally high levels of satisfaction with the customized orthosis. Participants reported favorable perceptions regarding comfort, fit, and ease of use. No adverse events or device-related complications were observed during the study period.

## Discussion

This study demonstrates that customized 3D-printed wrist-thumb orthoses are feasible in a clinical rehabilitation setting and are associated with meaningful reductions in pain and functional disability in patients with De Quervain’s tenosynovitis. Both the customized and conventional splints resulted in substantial clinical improvement, supporting the established role of immobilization as a first-line conservative intervention. Variability decreased over time, reflecting clinical convergence during recovery. The magnitude of pain reduction observed in both groups is consistent with contemporary evidence showing that wrist-thumb immobilization effectively reduces mechanical stress on the first dorsal compartment tendons, thereby alleviating pain and inflammation [14,15]. Recent systematic reviews and network meta-analyses have confirmed that splinting either alone or combined with other conservative measures remains an effective initial management strategy for De Quervain’s tenosynovitis [15,16].

Functional outcomes improved in both groups, with numerically greater improvement observed in the 3D-printed orthosis group, possibly due to enhanced anatomical conformity and optimized load distribution. However, no statistical comparisons were performed; therefore, no definitive conclusions regarding superiority can be drawn. Additive manufacturing allows precise control over orthosis geometry and thickness, enabling improved ergonomics and individualized fit, which are known determinants of adherence and functional recovery [1,4,22]. Similar benefits of customized orthoses have been reported in recent studies examining 3D-printed devices for upper-limb musculoskeletal conditions [22,23].

Patient satisfaction findings further support the clinical value of customization. High QUEST scores in the 3D-printed group reflect positive perceptions of comfort, ease of use, and overall device performance. Patient-reported satisfaction is increasingly recognized as a critical outcome in rehabilitation, as it influences compliance and long-term effectiveness of assistive devices [1,8]. The favorable satisfaction outcomes observed in this study align with recent reports indicating that patient-specific orthoses are better accepted than prefabricated alternatives [8,23]. Overall, the present study provides pragmatic clinical evidence supporting the integration of 3D printing technology into rehabilitation practice for De Quervain’s tenosynovitis, particularly in resource-constrained settings where patient-specific solutions may address limitations of conventional orthoses.

Future directions

Future research should focus on conducting adequately powered randomized controlled trials comparing customized 3D-printed orthoses with conventional splints in patients with De Quervain’s tenosynovitis. Studies incorporating longer follow-up periods are needed to evaluate the durability of clinical benefits, long-term adherence, and recurrence rates. Formal validation of the Bangla version of the QUEST 2.0 questionnaire would strengthen future patient-reported outcome assessments. In addition, cost-effectiveness analyses and implementation studies are warranted to determine the feasibility of integrating 3D-printing technology into routine rehabilitation practice, particularly in low-resource settings. Advances in digital scanning, computer-aided design, and additive manufacturing may further facilitate rapid production of patient-specific orthoses and support wider adoption of personalized rehabilitation interventions.

Limitations

This study has several limitations. The relatively small sample size and short duration of follow-up may restrict the generalizability of the findings and limit evaluation of long-term outcomes. Group allocation was not randomized and was determined by feasibility, which could have introduced selection bias and affected comparability between groups. Adherence to orthosis use was not objectively measured. In addition, the Bangla-translated version of the Quebec User Evaluation of Satisfaction with Assistive Technology (QUEST 2.0) used in this study has not undergone formal validation and should therefore be interpreted with caution. Moreover, individual-level data were not retained in a format suitable for reanalysis, preventing inferential statistical testing and independent verification of results. Furthermore, the cost-effectiveness of 3D-printed orthoses was not assessed in this study, and future economic evaluations are needed to determine their feasibility and value in routine clinical practice.

## Conclusions

Use of customized 3D-printed wrist-thumb orthoses is feasible in a rehabilitation setting and is associated with improvements in pain, functional outcomes, and patient satisfaction in patients with De Quervain’s tenosynovitis. Both conventional and customized orthoses demonstrated clinical benefit, yet the 3D-printed orthoses showed numerically greater improvements and higher patient satisfaction, probably due to enhanced anatomical conformity and ergonomics. These findings support the likely role of patient-specific orthoses in optimizing conservative management. Given the observational design, small sample size, and absence of inferential statistical analysis, these results should be interpreted as descriptive trends rather than definitive evidence of superiority. Future studies with larger sample sizes, randomized designs, and longer follow-up are warranted to confirm these findings and to evaluate cost-effectiveness and long-term clinical outcomes.
